# Radiofrequency Echographic Multi-Spectrometry (REMS) for Bilateral Femoral Neck Assessment in Pregnant Women: A Cross-Sectional Study

**DOI:** 10.3390/life16081249

**Published:** 2026-07-28

**Authors:** Elena Bischoff, Stoyanka Vladeva, Fabian Bischoff, Mira Hristova, Nikola Kirilov

**Affiliations:** 1 Department of Health Care, Faculty of Medicine, Trakia University, 6000 Stara Zagora, Bulgaria; 2 Rheumazentrum Ruhrgebiet Herne, Ruhr-Universität Bochum, 44649 Herne, Germany; 3 Rheumatology Practice Stara Zagora, 6000 Stara Zagora, Bulgaria; 4 Department of Obstetrics and genecology, Faculty of Medicine, Trakia University, 6000 Stara Zagora, Bulgaria; 5 Department of Orthopedics and Traumatology, University Hospital UMBAL Georgi Stranski, Medical University of Pleven, 5803 Pleven, Bulgaria

**Keywords:** pregnancy, bone mineral density, radiofrequency echographic Multi-Spectrometry, REMS, osteoporosis, femoral neck, ultrasound densitometry

## Abstract

Pregnancy is associated with physiological adaptations in calcium metabolism and skeletal homeostasis that may result in temporary reductions in bone mineral density (BMD). However, routine skeletal assessment during pregnancy remains limited because dual-energy X-ray absorptiometry (DXA) involves ionizing radiation. This study aimed to compare bilateral femoral neck BMD and Z-scores in pregnant and non-pregnant women using Radiofrequency Echographic Multi-Spectrometry (REMS), a radiation-free ultrasound-based technology, and to assess correlation between bilateral measurements. In this cross-sectional study, 100 women were enrolled, including 40 pregnant women and 60 non-pregnant women with comparable age and body mass index (BMI)**.** Bilateral femoral neck BMD and age-adjusted Z-scores were evaluated using REMS. Pregnant women had a mean age of 33 ± 5 years, pre-pregnancy BMI of 25.8 ± 6.5 kg/m^2^**,** and gestational age of 21 ± 5 weeks, with no significant differences in age or pre-pregnancy BMI compared with controls. Pregnant women demonstrated significantly lower left femoral neck BMD than controls (0.805 vs. 0.862 g/cm^2^; *p* = 0.0018) and lower left femoral neck Z-scores (−0.08 vs. 1.18 SD; *p* = 0.003). A strong positive correlation between left and right femoral neck BMD was observed (R = 0.78). Similarly, right femoral neck BMD was significantly lower in pregnant women compared with controls (0.835 g/cm^2^ vs. 0.892 g/cm^2^; *p* = 0.0026). The right femoral neck Z-score was 0.15 ± 1.15 SD in pregnant women and 1.45 ± 1.10 SD in controls (*p* = 0.002). REMS demonstrated the ability to detect differences in femoral neck bone parameters between pregnant and non-pregnant women while providing bilateral measurement consistency. These findings support the feasibility of REMS as a safe, radiation-free approach for assessing maternal skeletal status during pregnancy. However, the cross-sectional design and lack of longitudinal follow-up limit conclusions regarding the progression and reversibility of pregnancy-associated bone changes. Future prospective studies with larger cohorts and postpartum monitoring are needed to further define the clinical role of REMS in maternal bone health assessment.

## 1. Introduction

### 1.1. Bone Metabolism During Pregnancy

Pregnancy is characterized by major physiological adaptations affecting multiple organ systems, including the skeletal system. The developing fetus requires substantial amounts of calcium for skeletal mineralization, particularly during the second and third trimesters when fetal bone formation accelerates. It has been estimated that approximately 25–30 g of calcium are transferred from the mother to the fetus throughout pregnancy, with nearly 80% of this transfer occurring during the final trimester [1].

To meet these increasing calcium demands, maternal physiology undergoes several adaptive changes. Intestinal calcium absorption increases significantly under the influence of elevated concentrations of calcitriol, while alterations in parathyroid hormone-related peptide (PTHrP), estrogen, prolactin, and other hormones contribute to the regulation of mineral metabolism. Despite these compensatory mechanisms, maternal bone resorption may increase, resulting in measurable reductions in BMD [2].

Numerous studies have demonstrated transient decreases in maternal bone mass during pregnancy and lactation. Although these reductions are usually reversible after weaning, some women may experience excessive bone loss, predisposing them to fragility fractures and the rare but clinically significant condition known as pregnancy- and lactation-associated osteoporosis (PLO) [3,4,5,6]. Pregnancy-associated osteoporosis is considered a rare disorder, with an estimated incidence of approximately 0.4 per 100,000 pregnancies, although the true prevalence is likely underestimated because many cases remain undiagnosed or are diagnosed only after fragility fractures occur. The condition most commonly presents during the third trimester or early postpartum period with severe back pain, vertebral compression fractures and substantial impairment of quality of life. Early identification of women at risk is therefore of considerable clinical importance to prevent fractures and optimize maternal health while minimizing potential consequences for both mother and infant [4].

### 1.2. Challenges in Assessing Bone Health During Pregnancy

Assessment of skeletal status during pregnancy remains challenging. Dual-energy X-ray absorptiometry (DXA) is widely accepted as the gold standard for measuring BMD and diagnosing osteoporosis. However, concerns regarding fetal exposure to ionizing radiation generally preclude its routine use during pregnancy. Consequently, clinicians often lack objective tools for evaluating bone health in pregnant women who present with risk factors for osteoporosis, inflammatory diseases, endocrine disorders, prolonged immobilization, glucocorticoid exposure, malnutrition or previous fractures. Current clinical assessment therefore relies largely on the identification of risk factors, clinical history, biochemical markers of bone metabolism and imaging only when clinically indicated. Because no routine screening strategy for osteoporosis during pregnancy currently exists, the availability of a safe, radiation-free imaging technique could substantially improve the evaluation of women at increased skeletal risk. The development of radiation-free technologies capable of accurately evaluating skeletal status therefore represents a significant clinical need [7].

### 1.3. Radiofrequency Echographic Multi-Spectrometry (REMS)

REMS is an innovative ultrasound-based technology introduced during the last decade for the assessment of bone status at the lumbar spine and proximal femur. Unlike conventional quantitative ultrasound techniques that evaluate peripheral skeletal sites, REMS analyzes raw, unfiltered radiofrequency ultrasound signals obtained during scans of clinically relevant axial skeletal regions. Proprietary algorithms automatically identify bone interfaces and compare acquired spectral profiles with reference models derived from large populations, allowing the estimation of bone mineral density (BMD), T-scores, Z-scores and diagnostic classification [8,9].

In contrast to DXA, REMS does not use ionizing radiation, making it particularly attractive for patient populations in whom radiation exposure should be avoided, including pregnant women. The automated analysis and spectral approach may also reduce the influence of artifacts such as degenerative changes, calcifications and structural abnormalities that can affect conventional densitometric measurements. REMS provides a rapid, non-invasive and operator-independent assessment of skeletal status with measurements of the femoral neck and lumbar spine obtainable within a short examination time [10,11,12].

Several multicenter validation studies have demonstrated excellent agreement between REMS and DXA for BMD assessment at the lumbar spine and femoral neck with high diagnostic accuracy, strong correlation coefficients and excellent intra- and inter-operator precision and reproducibility. Furthermore, REMS has demonstrated potential for osteoporosis identification, fracture risk assessment and short-term monitoring of bone changes. These characteristics make REMS a promising imaging modality for evaluating bone health during pregnancy, where conventional DXA cannot be routinely performed [13,14,15,16,17]. Importantly, the feasibility of REMS assessment during pregnancy was explored by Degennaro et al. in a prospective case–control study involving 78 pregnant women with uncomplicated pregnancies (mean gestational age 39.1 ± 1.5 weeks). In this study, femoral neck BMD values obtained by REMS were compared with those of age- and pre-pregnancy BMI-matched non-pregnant controls. The authors demonstrated significantly lower femoral neck BMD values in pregnant women compared with controls (0.769 ± 0.094 g/cm^2^ vs. 0.831 ± 0.101 g/cm^2^; *p* = 0.0001), corresponding to an 8.1% reduction in BMD [18].

### 1.4. Rationale of the Study

Although initial evidence supports the feasibility of REMS for evaluating maternal bone status, data regarding its application during pregnancy remain limited. Degennaro et al. demonstrated lower femoral neck BMD values in women assessed at term pregnancy using REMS compared with matched non-pregnant controls. However, their study evaluated women only in late gestation and several aspects of maternal skeletal assessment using REMS remain insufficiently explored. In particular, the potential changes in femoral neck BMD earlier during pregnancy, the assessment of bilateral femoral neck measurements and the consistency between right and left proximal femur measurements have not been extensively investigated. The present study was therefore designed to assess femoral neck BMD and Z-score values in women during early and mid-pregnancy compared with non-pregnant women and to evaluate the bilateral relationship between left and right femoral neck measurements obtained using REMS technology.

## 2. Materials and Methods

### 2.1. Study Design

This cross-sectional study was conducted at tertiary rheumatology and orthopedic centers in Bulgaria between August 2023 and April 2026. The recruitment period was extended due to the inclusion of pregnant women meeting the predefined eligibility criteria and the need to obtain an adequate study sample within the specified inclusion criteria. All participants underwent bilateral femoral neck REMS assessment according to the study protocol. The study aimed to investigate differences in femoral neck BMD between pregnant and non-pregnant women using REMS, a novel radiation-free ultrasound-based technology. Given the inability to routinely perform DXA during pregnancy because of concerns regarding fetal exposure to ionizing radiation, REMS was selected as a safe alternative for the evaluation of skeletal status in this population [3,4,5].

The study was designed to compare femoral neck BMD and Z-score values between pregnant women and a non-pregnant control group with comparable age and BMI. Pre-pregnancy BMI was calculated using the weight recorded before pregnancy and the measured height (kg/m^2^) and was used to characterize baseline body composition and ensure comparability between study groups. Current gestational BMI was not used for group comparison because pregnancy-related weight gain reflects physiological changes, including increased fetal weight, plasma volume expansion and fluid retention, rather than baseline adiposity. In addition, bilateral femoral neck measurements were obtained to evaluate the correlation between the left and right proximal femur and to explore the feasibility of bilateral REMS assessment in pregnant and non-pregnant women.

### 2.2. Study Population

A total of 100 women were enrolled in the study and allocated into two groups according to pregnancy status. The study group consisted of 40 pregnant women (40% of the total study population), who were recruited during routine obstetric follow-up visits. The control group consisted of 60 healthy non-pregnant women (60% of the study population) recruited from the same geographical region and assessed during the same study period as the pregnant participants. Controls were selected to be comparable with the pregnant group regarding age and pre-pregnancy BMI.

### 2.3. Inclusion and Exclusion Criteria

Pregnant women were eligible for inclusion if they were aged 18 years or older, had a singleton pregnancy and attended routine antenatal follow-up visits during the study period. Healthy non-pregnant women aged 18 years or older from the same geographical region were eligible for inclusion as controls.

Participants in both groups were excluded if they had a known metabolic bone disease, previous fragility fracture, thyroid or parathyroid disorders, chronic kidney disease, malabsorption syndromes (including celiac disease), inflammatory rheumatic diseases or any other chronic disorder known to affect bone metabolism. Women receiving medications known to influence bone health, including systemic glucocorticoids, anti-osteoporotic therapy, anticonvulsants or other bone-active medications, were also excluded. In the pregnant group, women with multiple (twin or higher-order) pregnancies were excluded. Participants were additionally excluded if REMS assessment could not be performed according to the standardized study protocol.

### 2.4. REMS Assessment

REMS examinations were performed using the EchoStudio echographic device (Echolight S.p.a., Lecce, Italy), equipped with a convex transducer operating at a central frequency of 3.5 MHz. During examination, the transducer was positioned over the hip region to visualize the predefined regions of interest (ROI). After acquisition of an adequate B-mode ultrasound image, the REMS software (version 2.2.1) automatically identified the bone interfaces at the target skeletal sites. Ultrasound frame sequences were continuously recorded for approximately 40 s for the proximal femur, allowing automated identification and analysis of the ROI.

Unlike conventional ultrasound techniques, which rely primarily on image interpretation, REMS analyzes the spectral characteristics of radiofrequency signals reflected by bone tissue. These signals are compared with reference spectral models derived from large databases of healthy and osteoporotic subjects, allowing the calculation of BMD values and diagnostic indices [9].

The REMS examination consisted of the following sequential steps:

1. Patient positioning and preparation.
2. Ultrasound localization of the proximal femur.
3. Identification of the femoral neck region of interest.
4. Acquisition of native radiofrequency ultrasound signals.
5. Spectral analysis of the acquired signals.
6. Comparison with validated reference spectral models.
7. Automatic calculation of BMD and diagnostic parameters.

**Figure 1:**
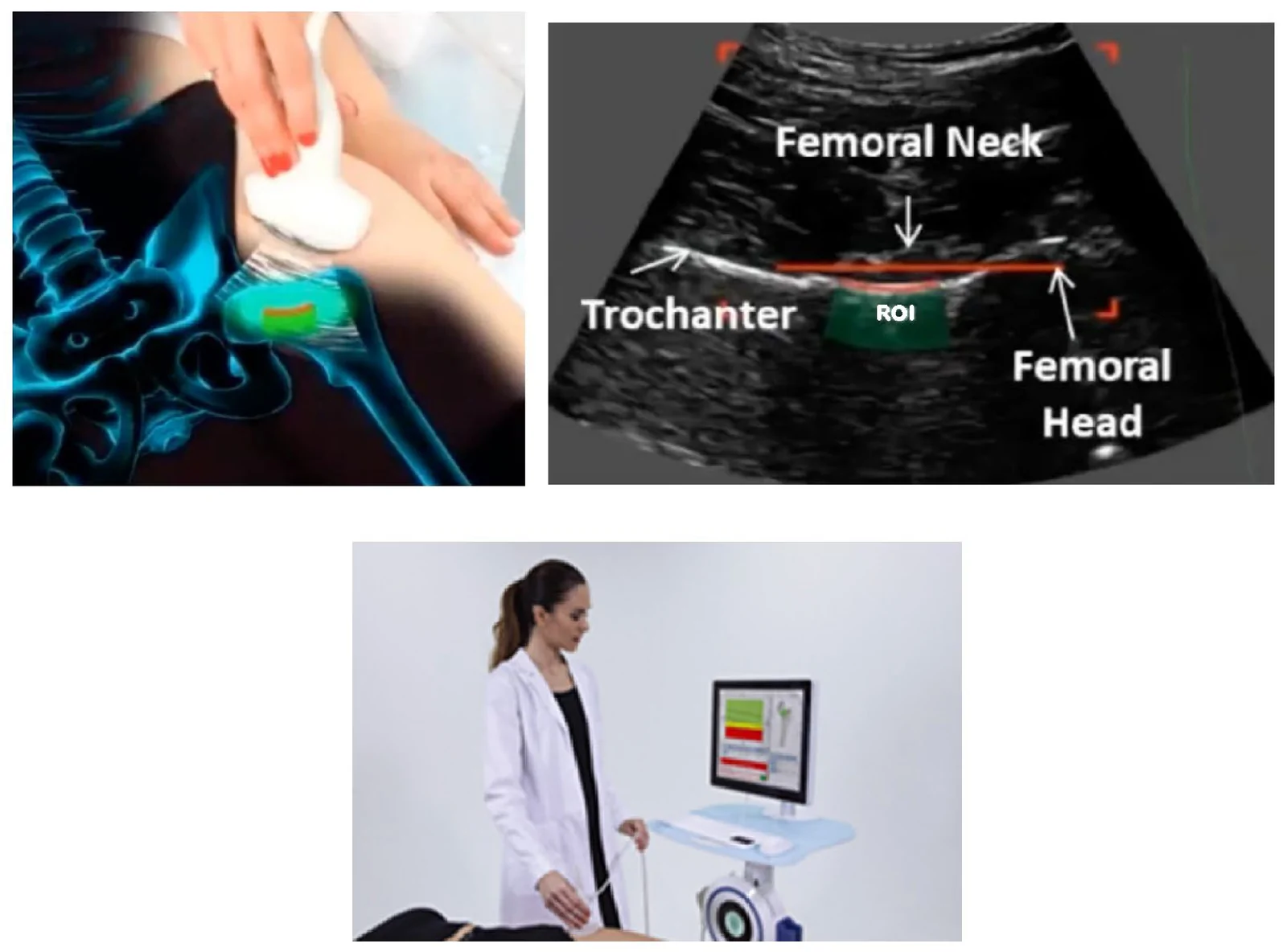
Illustration of REMS assessment of the hip.

All REMS examinations were performed by a single experienced operator, and the measurements were performed for both the left and right femoral neck in all participants following a standardized scanning protocol. Bilateral assessment was performed to investigate potential differences between femora and to evaluate the consistency of REMS measurements at both skeletal sites (Figure 1).

### 2.5. Outcome Measures

The primary outcome measures of the study were REMS-derived femoral neck BMD and Z-score values.

The following parameters were recorded:

- Left femoral neck BMD (g/cm^2^)
- Right femoral neck BMD (g/cm^2^)
- Left femoral neck Z-score
- Right femoral neck Z-score

BMD values represented the quantitative estimate of bone mineral density generated by the REMS system. Z-scores represented the number of standard deviations by which an individual’s BMD differed from the mean value of an age-specific reference population.

Secondary outcome measures included:

- Correlation between left and right femoral neck BMD measurements
- Comparison of BMD values between pregnant and non-pregnant women
- Comparison of Z-score values between pregnant and non-pregnant women

### 2.6. Statistical Analysis

Statistical analyses were performed using SPSS software (version 19.0; IBM Corp., Armonk, NY, USA) to evaluate differences between groups and relationships among measured variables. Continuous variables were expressed as mean values ± standard deviation (SD), together with minimum and maximum values where appropriate.

The normality of data distribution was assessed before performing comparative analyses. Comparisons between pregnant and non-pregnant women were conducted using appropriate statistical tests according to the distribution of the variables under investigation. For normally distributed continuous variables, comparisons between the two independent groups were performed using the independent Student’s *t*-test. This test was used to determine whether there were statistically significant differences in mean values between pregnant and non-pregnant women. For variables not meeting the assumptions of normal distribution, non-parametric tests were applied as appropriate.

Pearson correlation analysis was performed to evaluate the relationship between left and right femoral neck BMD values in pregnant women. Pearson’s correlation coefficient (r) was calculated to quantify the strength and direction of the linear association between bilateral measurements. Correlation coefficients range from −1 to +1, with positive values indicating a direct relationship and values closer to ±1 representing stronger associations. Correlation coefficients were calculated to assess the strength and direction of the association between bilateral measurements. Paired *t*-tests were used to assess differences between right and left femoral neck BMD values, as these measurements were obtained from the same individuals. This analysis was performed to determine whether there were statistically significant side-to-side differences in femoral neck BMD. A *p*-value below 0.05 was considered statistically significant for all analyses. Statistical significance was interpreted as evidence against the null hypothesis and indicated a meaningful difference or association between the examined variables.

### 2.7. Ethical Considerations

This study was conducted in accordance with the Declaration of Helsinki and approved by the ethics committee for scientific research of the Medical Faculty, Trakia University, Stara Zagora, Bulgaria (Protocol Number: 26, date 1 June 2023).

## 3. Results

### 3.1. Baseline Characteristics

The mean age of the pregnant women was 33 ± 5 years, with an age range between 25 and 42 years. The mean pre-pregnancy BMI was 25.8 ± 6.5 kg/m^2^, ranging from 15 to 41 kg/m^2^.

The mean gestational age at the time of examination was 21 ± 5 weeks, with a range between 13 and 28 weeks of gestation. Therefore, all participants were examined during the first or second trimester, with the majority assessed during the second trimester. No participants were evaluated during the third trimester.

The mean age of the control participants was 31 ± 6 years, ranging from 24 to 40 years. The mean BMI was 24.7 ± 5.3 kg/m^2,^ with values ranging between 16 and 36 kg/m^2^.

The absence of major differences in age and BMI between the two groups minimized the potential influence of these important determinants of BMD and allowed a more reliable comparison of skeletal parameters.

No substantial differences in age or BMI were observed between pregnant and non-pregnant participants, supporting comparability of the study groups (Table 1).

### 3.2. Femoral Neck BMD Assessment

Femoral neck BMD was assessed in pregnant women using REMS. In this group, the left femoral neck BMD was 0.805 ± 0.160 g/cm^2^, while the right femoral neck BMD was 0.835 ± 0.150 g/cm^2^. When compared with non-pregnant controls, the mean left femoral neck BMD was significantly lower in pregnant women (0.805 g/cm^2^ vs. 0.862 g/cm^2^; *p* = 0.0018). Similarly, right femoral neck BMD was significantly lower in pregnant women compared with controls (0.835 g/cm^2^ vs. 0.892 g/cm^2^; *p* = 0.0026). Regarding femoral neck Z-scores, both measurements demonstrated lower values in pregnant women compared with non-pregnant controls. The right femoral neck Z-score was 0.15 ± 1.15 SD in pregnant women and 1.45 ± 1.10 SD in controls (*p* = 0.002), while the left femoral neck Z-score was −0.08 ± 1.15 SD and 1.18 ± 1.10 SD, respectively (*p* = 0.003) (Table 2).

Compared with non-pregnant controls, pregnant women demonstrated a 6.6% lower left femoral neck BMD (0.805 vs. 0.862 g/cm^2^) and a 6.4% lower right femoral neck BMD (0.835 vs. 0.892 g/cm^2^). Regarding femoral neck Z-scores, pregnant women showed an 89.7% lower right femoral neck Z-score (0.15 vs. 1.45 SD) and a 106.8% lower left femoral neck Z-score (−0.08 vs. 1.18 SD) compared with non-pregnant controls.

**Figure 2:**
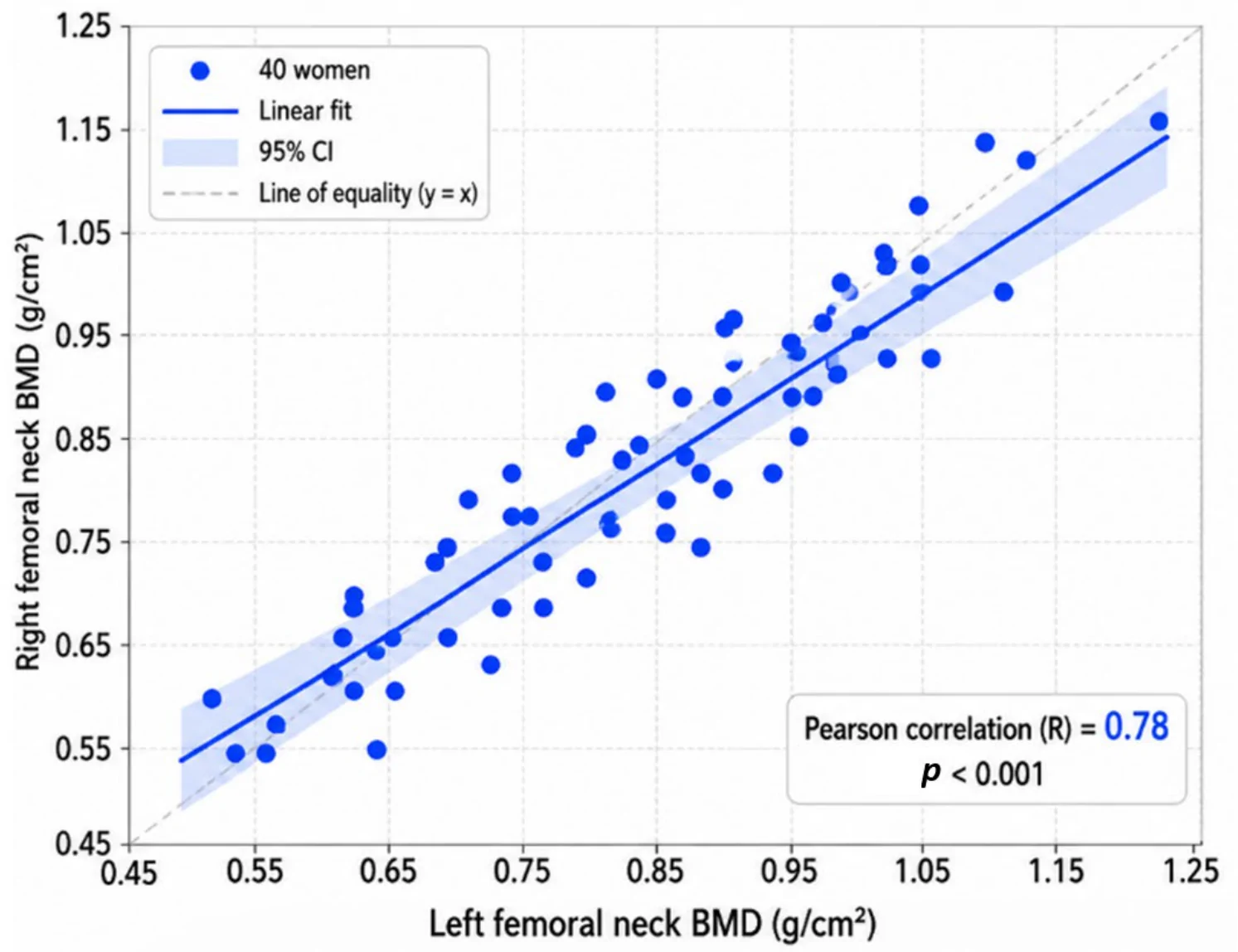
Correlation between left and right femoral neck BMD measurements in pregnant women.

A strong positive correlation was observed between left and right femoral neck BMD measurements in both study groups. In pregnant women, the Pearson correlation coefficient was r = 0.78 (95% CI: 0.63–0.88; *p* < 0.001), while in non-pregnant women, the correlation coefficient was r = 0.82 (95% CI: 0.71–0.89; *p* < 0.001). The correlation analysis comparing bilateral femoral neck measurements in pregnant women is presented in Figure 2.

The scatter plot demonstrates the relationship between left and right femoral BMD values measured by REMS in 40 pregnant women. Each dot represents an individual participant. The x-axis shows the left femoral neck BMD values (g/cm^2^), while the y-axis represents the corresponding right femoral neck BMD values (g/cm^2^).

A strong positive linear association was observed between bilateral femoral neck measurements, as demonstrated by the fitted regression line (solid blue line). The shaded area represents the 95% confidence interval (CI) of the regression estimate. The dashed diagonal line represents the line of equality (y = x), where identical values between the left and right femoral neck would be expected.

The Pearson correlation coefficient was R = 0.78 (*p* < 0.001), indicating a strong and statistically significant correlation between left and right femoral neck BMD measurements. The findings demonstrate a high degree of bilateral correlation between femoral neck measurements obtained by REMS in pregnant women, suggesting consistent assessment of skeletal parameters between the two sides.

A paired comparison between right and left femoral neck BMD values was performed to assess potential side-to-side differences. Although the right femoral neck BMD values were slightly higher than the left femoral neck values in both groups, the differences were not statistically significant (pregnant women: *p* = 0.08; non-pregnant women: *p* = 0.06).

## 4. Discussion

### 4.1. Main Findings

The present study demonstrates that pregnant women exhibit significantly lower femoral neck BMD and Z-score values compared with a non-pregnant control group with comparable age and BMI. This reduction was observed bilaterally, with a strong correlation between left and right femoral neck measurements, indicating bilateral anatomical consistency of femoral neck BMD values. These findings provide direct evidence that pregnancy is associated with measurable alterations in maternal skeletal status. Importantly, our study confirms the feasibility of REMS as a safe, radiation-free and clinically practical tool for assessing bone health during pregnancy. Unlike DXA, which remains the reference technique for osteoporosis diagnosis but involves exposure to ionizing radiation and is therefore generally avoided during pregnancy, REMS uses ultrasound-based radiofrequency analysis and allows repeated skeletal assessments without radiation exposure. This characteristic is particularly relevant in pregnancy, where longitudinal monitoring of maternal bone changes may be clinically desirable but limited by safety considerations.

The reduction in BMD observed in our cohort is clinically relevant, even if transient, because it may predispose susceptible individuals to future osteoporosis or fractures, particularly in women with preexisting risk factors. Identifying these changes early provides an opportunity for timely nutritional and lifestyle interventions, as well as risk stratification for high-risk populations.

### 4.2. Comparison with Previous Studies

Our findings align with prior investigations reporting reductions in maternal BMD during pregnancy and lactation. Kovacs and colleagues described significant physiological adaptations in calcium metabolism, emphasizing the transient nature of skeletal loss associated with reproductive events [19]. Degennaro et al. were among the first to evaluate maternal bone status during pregnancy using REMS and reported significantly lower femoral neck BMD values in pregnant women compared with age- and pre-pregnancy BMI-matched non-pregnant controls. In their cohort of 78 women assessed at term pregnancy (mean gestational age 39.1 ± 1.5 weeks), mean femoral BMD was 0.769 ± 0.094 g/cm^2^ in pregnant women compared with 0.831 ± 0.101 g/cm^2^ in controls, corresponding to an 8.1% reduction in BMD. In our study, women were assessed earlier in pregnancy, predominantly during the second trimester (mean gestational age 21 ± 5 weeks), and similarly demonstrated significantly lower femoral neck BMD values compared with non-pregnant controls. The observed reduction was 6.6% for the left femoral neck (0.805 vs. 0.862 g/cm^2^) and 6.4% for the right femoral neck (0.835 vs. 0.892 g/cm^2^). Although the absolute BMD values and magnitude of reduction differed between studies, these findings consistently demonstrate the ability of REMS to identify lower femoral bone parameters in pregnant women. The slightly lower reduction observed in our cohort may be related to the earlier gestational age at assessment, as Degennaro et al. evaluated women at the end of pregnancy, when fetal calcium demand and maternal skeletal adaptations are expected to be greater [18].

Multiple validation studies have confirmed that REMS measurements strongly correlate with DXA, showing high precision and reproducibility across skeletal sites.

The diagnostic performance and precision of REMS have been extensively investigated in comparison with DXA. Multiple validation studies have reported strong correlations between REMS-derived BMD values and DXA measurements, with Pearson correlation coefficients generally ranging from approximately 0.90 to 0.97 across different skeletal sites, demonstrating a high degree of agreement between the two methods. Furthermore, studies evaluating the ability of REMS to identify osteoporosis have reported diagnostic accuracy values with sensitivity and specificity frequently exceeding 85–90%, suggesting that REMS may reliably discriminate individuals with reduced bone mass.

Measurement precision represents another important consideration when evaluating a technique intended for monitoring bone changes over time. REMS has demonstrated excellent short-term reproducibility with coefficients of variation (CV) generally below 1%, while DXA precision typically ranges between approximately 1–2% depending on skeletal site and device characteristics. Reported least significant change (LSC) values for REMS are approximately 1% at the lumbar spine and femoral neck, which are substantially lower than commonly reported DXA LSC thresholds of approximately 3–5%. These characteristics suggest that REMS may be particularly suitable for detecting relatively small longitudinal changes in bone parameters.

Therefore, the clinical value of REMS may be greatest in situations where repeated assessment is needed or where DXA is unsuitable such as pregnancy, pediatric populations or frequent monitoring scenarios [13,14,15,16,17].

In the present study, the correlation between right and left femoral neck measurements was strong in both pregnant women (r = 0.78, 95% CI: 0.63–0.88) and non-pregnant controls (r = 0.82, 95% CI: 0.71–0.89), confirming bilateral consistency of femoral neck assessment. These findings suggest that unilateral measurements may adequately represent femoral neck bone status; however, bilateral evaluation may provide additional confidence in specific clinical or research settings.

### 4.3. Clinical Implications

REMS offers a valuable tool for identifying women at risk for pregnancy-associated bone loss, especially in populations with additional risk factors. These include women with autoimmune rheumatic diseases, patients receiving long-term glucocorticoids, women with inflammatory bowel disease, individuals with prior fragility fractures, patients with nutritional deficiencies and those with suspected pregnancy-associated osteoporosis.

The ability to perform repeated, radiation-free assessments allows clinicians to monitor skeletal changes longitudinally throughout pregnancy and into the postpartum period. Early detection of significant bone loss may guide interventions such as dietary optimization (calcium and vitamin D supplementation), weight-bearing exercise and targeted pharmacological therapy when indicated. In addition, REMS could serve as a research tool to better understand the trajectory of skeletal changes in different maternal populations and inform public health guidelines on maternal bone health.

### 4.4. Strengths

This study has several notable strengths. First, it utilizes an innovative, radiation-free imaging modality, making it suitable for use in pregnant populations. Second, the evaluation of both femoral necks allows assessment of bilateral correlation, increasing confidence in measurement reliability. Third, the inclusion of a non-pregnant control group with comparable age and BMI strengthens the validity of the comparisons while reducing the potential influence of these important confounding variables. Finally, this study examines a clinically understudied population, providing valuable data for maternal skeletal health research and clinical practice.

### 4.5. Limitations

Despite these strengths, several limitations should be acknowledged. The relatively small sample size and exploratory design represent important limitations, and no a priori sample size calculation was performed; therefore, the findings require confirmation in larger prospective studies. Multivariable analysis was not performed, and potential confounding factors such as age, BMI, parity, calcium and vitamin D supplementation, physical activity and nutritional status could not be fully evaluated. The cross-sectional design and absence of biochemical bone turnover markers limit the interpretation of skeletal changes over time. Additionally, the absence of pre-pregnancy BMD measurements represents a limitation as baseline individual bone status and the magnitude of pregnancy-related changes could not be assessed. Furthermore, the absence of postpartum BMD follow-up limited the assessment of skeletal recovery after pregnancy and lactation. The lack of a population-specific Bulgarian REMS reference database and the inclusion of participants at different gestational stages may also have influenced the interpretation of bone parameters. Future longitudinal studies incorporating larger cohorts, pre-pregnancy baseline assessments, postpartum monitoring and additional clinical and biochemical parameters are needed to further characterize bone changes during pregnancy and lactation.

## 5. Conclusions

Pregnant women in this study demonstrated significantly lower femoral neck BMD and Z-score values than non-pregnant controls. Bilateral REMS measurements showed a strong correlation, indicating consistency between right and left femoral neck measurements and reflecting bilateral anatomical symmetry. These findings suggest that REMS is a feasible, radiation-free method for assessing bone health during pregnancy. However, given the pilot, cross-sectional design, the limited sample size and the absence of longitudinal follow-up, the present results should be interpreted with caution. Further large-scale, prospective studies are needed to validate these findings, determine the clinical utility of REMS during pregnancy and clarify its potential role in the assessment and monitoring of maternal bone health.

## Figures and Tables

**Table 1:** Demographic Characteristics.

Variable	Pregnant Women	Non-Pregnant Women	*p*-Value
Number of participants	40	60	—
Age (years)	33 ± 5 (95% CI: 31.4–34.6)	31 ± 6 (95% CI: 29.5–32.5)	0.18 (NS)
Pre-pregnancy BMI (kg/m^2^)	25.8 ± 6.5 (95% CI: 23.7–27.9)	24.7 ± 5.3 (95% CI: 23.3–26.1)	0.27 (NS)
Gestational age (weeks)	21 ± 5 (95% CI: 19.4–22.6)	N/A	—

**Table 2:** Femoral Neck Bone Mineral Density and Z-scores.

Parameter	Pregnant Women	Non-Pregnant Women	*p*-Value
Left femoral neck BMD (g/cm^2^)	0.805 ± 0.160 (95% CI: 0.754–0.856)	0.862 ± 0.148 (95% CI: 0.824–0.900)	0.0018
Right femoral neck BMD (g/cm^2^)	0.835 ± 0.150 (95% CI: 0.787–0.883)	0.892 ± 0.148 (95% CI: 0.854–0.930)	0.0026
Right femoral neck Z-score (SD)	0.15 ± 1.15 (95% CI: −0.22–0.52)	1.45 ± 1.10 (95% CI: 1.17–1.73)	0.002
Left femoral neck Z-score (SD)	−0.08 ± 1.15 (95% CI: −0.45–0.29)	1.18 ± 1.10 (95% CI: 0.90–1.46)	0.003
Right–Left correlation (R)	r = 0.78 (95% CI: 0.63–0.88)	r = 0.82 (95% CI: 0.71–0.89	<0.001

## Data Availability

The authors confirm that the data supporting the findings of this study are not publicly available due to privacy and ethical restrictions.
